# Supplementary material for: Resolving the full spectrum of human genome variation using Linked-Reads
Source: Genome Res. 2019 Apr;29(4):635–45. doi: 10.1101/gr.234443.118 (PMC6442396; doi:10.1101/gr.234443.118)
Supplement: Supplemental Material [file supp_gr.234443.118_Supplemental_longranger-2.2.zip › longranger-2.2.tar.bz2!/lr2.2-public/lib/stan/cmdstan/stan/doc/api/html/math_2matrix_2sum_8hpp_source.html]

Stan: src/stan/math/matrix/sum.hpp Source File


|  |  |
| --- | --- |
|  | Stan  2.5.0  probability, sampling & optimization |


- Main Page
- Related Pages
- Namespaces
- Classes
- Files

- File List
- File Members

All Classes Namespaces Files Functions Variables Typedefs Enumerations Enumerator Friends Macros Pages

- src
- stan
- math
- matrix

sum.hpp

Go to the documentation of this file.

1 #ifndef STAN\_\_MATH\_\_MATRIX\_\_SUM\_HPP

2 #define STAN\_\_MATH\_\_MATRIX\_\_SUM\_HPP

3

4 #include <vector>

5 #include <stan/math/matrix/Eigen.hpp>

6

7 namespace stan {

8  namespace math {

9

18  template <typename T>

19  inline T sum(const std::vector<T>& xs) {

20  if (xs.size() == 0) return 0;

21  T sum(xs[0]);

22  for (size\_t i = 1; i < xs.size(); ++i)

23  sum += xs[i];

24  return sum;

25  }

26

33  template <typename T, int R, int C>

34  inline double sum(const Eigen::Matrix<T,R,C>& v) {

35  return v.sum();

36  }

37

38  }

39 }

40 #endif

Eigen.hpp

stan::math::sum

double sum(std::vector< double > &x)

**Definition:** sum.hpp:10


---

[ Stan Home Page ]

*© 2011–2014,
Stan Development Team.*
